# CRISPR Screens Identify *PIK3C2A* as a Novel Mediator of EGFR Inhibitor Resistance in Head and Neck Squamous Cell Carcinoma

**DOI:** 10.1002/hed.70048

**Published:** 2025-09-23

**Authors:** Jiayu Wang, Megan L. Ludwig, Aditi Kulkarni, Andrew C. Birkeland, Nicole L. Michmerhuizen, Elizabeth Gensterblum‐Miller, Jingyi Zhai, Hui Jiang, Paul Swiecicki, Marisa Buchakjian, Molly Heft Neal, Steven B. Chinn, Matthew E. Spector, J. Chad Brenner

**Affiliations:** ^1^ Department of Pharmacology University of Michigan Ann Arbor Michigan USA; ^2^ Department of Otolaryngology—Head and Neck Surgery University of Michigan Ann Arbor Michigan USA; ^3^ Cellular and Molecular Biology Program University of Michigan Ann Arbor Michigan USA; ^4^ Department of Otolaryngology—Head and Neck Surgery University of California Davis California USA; ^5^ Department of Biostatistics University of Michigan Ann Arbor Michigan USA; ^6^ Rogel Cancer Center University of Michigan Ann Arbor Michigan USA; ^7^ Department of Hematology Oncology University of Michigan Ann Arbor Michigan USA; ^8^ University of Pittsburgh Medical Center University of Pittsburgh Pittsburgh Pennsylvania USA

**Keywords:** CRISPR, drug resistance, EGFR inhibitor, HNSCC, PIK3C2A

## Abstract

**Background:**

Head and neck squamous cell carcinoma (HNSCC) is a malignancy with poor prognosis and survival. While epidermal growth factor receptor (EGFR) is a known driver of HNSCC, EGFR inhibitors show limited efficacy as monotherapies, suggesting that effective combination therapies are needed.

**Methods:**

We performed GeCKO and Kinase CRISPR library screens to identify candidate knockouts that increase EGFR inhibitor sensitivity in resistant HNSCC cells. We validated a candidate using the CellTiter‐Glo viability assay following RNA interference and investigated the mechanisms using apoptosis and cell cycle flow cytometry analysis.

**Results:**

Kinase CRISPR library screens identified that *PIK3C2A* gene downregulation enhanced EGFR inhibitor sensitivity in resistant HNSCC cells. Viability assays further validated that *PIK3C2A* knockdown overcame EGFR inhibitor resistance in HNSCC cells.

**Conclusions:**

Our data suggests that *PIK3C2A* is a novel mediator of EGFR inhibitor resistance in HNSCC. Future studies are required to determine the mechanisms by which *PIK3C2A* drives EGFR inhibitor resistance.

## 1 Introduction

Head and neck squamous cell carcinoma (HNSCC) accounts for approximately 90% of all head and neck cancers. HNSCC remains among the top 10 most common cancers worldwide, with an estimated 890 000+ new cases and 480 000+ deaths reported in 2022, according to GLOBOCAN 2022 [1]. Surgery followed by adjuvant radiation or definitive chemoradiation is a key treatment strategy for primary HNSCC treatment depending on the tumor subsite and disease stage. Despite advancements in treatments, locoregionally advanced disease, metastasis, high recurrence rates, and poor survival remain prevalent among HNSCC patients [2, 3]. Although the immune checkpoint inhibitors nivolumab and pembrolizumab showed clinically significant improvements in survival for patients with recurrent and/or metastatic (R/M) HNSCC, the response rate for these two drugs is limited to only 13%–18% [4, 5, 6, 7].

Previous studies have shown that the epidermal growth factor receptor (EGFR) and its ligand, transforming growth factor alpha (TGF‐α), are prevalently overexpressed, with elevated mRNA levels detected in 92% and 87.5% of HNSCC tumors, respectively [8]. Additionally, the overexpression of EGFR and TGF‐α is correlated with reduced survival in HNSCC patients [9]. Upon activation, EGFR initiates downstream signaling pathways, including the phosphatidylinositol 3‐kinase (PI3K)/protein kinase B (AKT) and RAS/MEK/ERK pathways, to drive tumor survival, proliferation, angiogenesis, invasion, and metastasis [10, 11]. These data highlight EGFR as a promising therapeutic target for the treatment of HNSCC. Indeed, the EGFR inhibitor cetuximab in combination with radiation enhanced locoregional control and prolonged patient survival in locoregionally advanced HNSCC and has been FDA‐approved for this patient population [12]. In 2011, the FDA also approved the combination of cetuximab with platinum‐based chemotherapy and 5‐fluorouracil as a first‐line treatment for R/M HNSCC [13]. However, cetuximab as a monotherapy showed limited efficacy, with a response rate of only 13% [14]. A clinical trial in patients with human papilloma positive oropharyngeal carcinoma also revealed that radiotherapy plus cetuximab resulted in inferior survival compared to radiotherapy plus cisplatin [15]. Another small‐molecule EGFR inhibitor, gefitinib, also showed unsatisfactory therapeutic efficacy in improving overall survival as a monotherapy in patients with R/M HNSCC [16]. Similarly, other clinical trials evaluating EGFR‐targeting agents, including afatinib, zalutumumab, and panitumumab, have shown limited success in HNSCC patients [17, 18, 19].

While monotherapies may be limited in efficacy, combination therapy—a multi‐dimensional treatment approach that combines two or more agents—can overcome drug resistance, improve effectiveness, and reduce toxicity as well as the risk of cancer recurrence [20, 21, 22, 23]. Although the intricate nature of cell signaling pathways makes the discovery of effective combinations laborious, the adaptation of the CRISPR/Cas9 genetic engineering platform for mammalian systems significantly simplifies the process of generating arrays of genetic knockouts, making identifying key drivers of drug resistance more feasible. By applying a therapy in cells treated with a pooled CRISPR library, we can simultaneously screen hundreds of genetic knockouts that enhance treatment sensitivity to identify potential targets for combination therapy [24, 25, 26].

In this study, we investigated the mechanisms of resistance to EGFR inhibitors in HNSCC using CRISPR libraries. We introduced genome‐wide and kinome‐wide CRISPR libraries in HNSCC cell line models resistant to EGFR inhibition and conducted screens to identify genetic knockouts that enhance sensitivity to gefitinib or erlotinib. *PIK3C2A* was the most consistent candidate across multiple cell lines. We further validated *PIK3C2A* as a driver of EGFR inhibitor resistance by CellTiter‐Glo viability assays and investigated how the combination of the EGFR inhibitor with *PIK3C2A* knockout impacts HNSCC viability.

## 2 Methods

### 2.1 Cell Culture

Human UM‐SCC and Detroit 562 cell lines were cultured in Dulbecco's Modified Eagle's Medium (DMEM) (Invitrogen, 11965) with 10% heat‐inactivated fetal bovine serum (Corning, MT35016CV), 1% non‐essential amino acids (Invitrogen, 15140122), and 7 μL/mL (1%) penicillin–streptomycin (Invitrogen, 15140122) in a humidified atmosphere of 5% CO_2_ at 37°C. Mycoplasma contamination was screened by using the MycoAlert detection kit (Lonza).

### 2.2 Chemicals

All compounds were initially dissolved in sterile DMSO and diluted in medium to the indicated concentrations. After integration of CRISPR libraries, cells were treated with 1 μM gefitinib (Selleckchem S1025) or 1 μM erlotinib (Selleckchem S7786) in triplicate. For the Kinase Library samples, the DMSO control was also in triplicate, while the GeCKO Library samples had one DMSO control treatment. For CellTiter‐Glo and flow cytometry analysis, cells were dosed with either DMSO or 1 μM gefitinib 24 h after transfection.

### 2.3 EGFR Resistance Analysis

Gefitinib IC50 values for non‐UM‐SCC cell lines were downloaded from The Genomics of Drug Sensitivity in Cancer Project (cancerrxgene.org) [27, 28, 29], using data version 17.3. Gefitinib IC50 value for Detroit 562 was further determined by using the resazurin cell viability assay.

### 2.4 Preparation and Analysis of CRISPR Libraries

Methods are available in Supporting Information: Methods.

### 2.5 Small Interfering RNA (siRNA) Transfection

About 100 000 cells (for 6‐well plates) or 10 000 cells (for 96‐well plates) were seeded. After 24 h, cells were treated with either DharmaFECT 1 transfection reagent only (Horizon, Catalog ID:T‐2001‐03), 25 nM ON‐TARGETplus non‐targeting control pool (NTP) (Horizon, Catalog: Catalog ID: D‐001810‐10‐20), or 25 nM ON‐TARGETplus *PIK3C2A* siRNA SMARTPool (Horizon, Catalog ID:L‐006771‐00‐0020). The siRNAs and transfection reagent were diluted in serum‐free DMEM medium and incubated at room temperature for 5 min. They were then combined (siRNA was added to transfection reagent) and incubated again at room temperature for 20 min. Antibiotic‐free complete DMEM was added to dilute the siRNAs to a final concentration of 25 nM. In the transfection reagent only control group, an equal volume of sterile water was used in place of the siRNA. Finally, 2 mL of siRNA‐containing transfection medium was added to cells in a 6‐well plate, or 100 μL was added to cells in a 96‐well plate.

### 2.6 Resazurin Cell Viability Assays

About 2000 cells per well were seeded in 384‐well microplates using a Multiflo liquid handling dispensing system. After 24 h, cells were treated with compounds or DMSO in a 10‐point two‐fold dilution series in quadruplicate. 96‐well plates were prepared with compounds in 200× concentration and then diluted to 10× concentration in media in a second 96‐well plate using the Agilent Bravo Automated Liquid Handling Platform and VWorks Automation Control Software. These compounds were then used to treat the cells with the desired drug concentration, again using liquid handling robotics. Cells were stained with 67.6 μM resazurin (Thermo Fisher Scientific, B21187.06) for 24 h before fluorescent signal intensity was quantified. After 72 h treatment, fluorescent signal was obtained using the Cytation3 fluorescence plate reader enabled with automatic stacking at excitation and emission wavelengths of 540 and 612 nm, respectively.

### 2.7 CellTiter‐Glo Viability Assay

At 24 h after transfection, DMSO (0.5% v/v) or 1 μM gefitinib was added to transfected cells in 96‐well plates (Corning, 3603). After 48 h, 100 μL of CellTiter‐Glo reagent was added to both the treatment wells and the control wells, which contained medium without cells. The CellTiter‐Glo reagent (Promega, G7572) was prepared according to the manufacturer's instructions. 96‐well plates were incubated on an orbital shaker at room temperature for 10 min to induce cell lysis. Luminescence data were subsequently collected using the Cytation 3 plate reader.

### 2.8 Annexin V Cell Apoptosis Assay

At 24 h after siRNA transfection, DMSO (0.5% v/v) or 1 μM gefitinib was added to transfected cells in 6‐well plates. After 48 h of drug treatment, the culture medium and PBS used for washing the wells were collected and centrifuged to collect the suspended cells. The attached cells were harvested using trypsin, and then both cell populations were combined in round‐bottom tubes. Cells were washed with cold PBS, centrifuged one more time, and then suspended in 100 μL 1× annexin binding buffer. Annexin binding buffer and 100 μg/mL propidium iodide (PI) were prepared according to the manufacturer's instructions (Invitrogen, V13242). Next, 1 μL 100 μg/mL PI and 5 μL FITC Annexin V were added to round‐bottom tubes, and cells were stained at room temperature for 15 min. After the 15 min incubation, 400 μL 1× annexin binding buffer was added to tubes, and samples were kept on ice prior to flow cytometry. Cells were analyzed by Bio‐Rad ZE5 Cell Analyzer or ThermoFisher Bigfoot Cell Sorter, and flow cytometry data was collected from the flow cytometry core at the University of Michigan.

### 2.9 Cell Cycle Flow Cytometry Analysis

Cells were treated following the same protocol described for the cell apoptosis assay. About 10 μM EdU was added to the cell culture during the last 2 h of treatment. Both suspended and attached cells were collected and washed with 1% BSA in PBS (Teknova, P1391). Cells were then fixed, permeabilized, and processed through the Click‐iT reaction following the manufacturer's instructions for the Click‐iT EdU Alexa Fluor 488 Flow Cytometry Assay Kit (Invitrogen, C10425). After the Click‐iT reaction, cells were washed and resuspended in 1 mL of 1× Click‐iT saponin‐based permeabilization and wash reagent. Subsequently, 1 μL FxCycle violet stain (Invitrogen, F10347) was added to each sample for an additional 30 min incubation at room temperature. Cells were analyzed by the Bio‐Rad ZE5 Cell Analyzer, and flow cytometry data were collected from the flow cytometry core at the University of Michigan.

### 2.10 qPCR

Cells were rinsed with PBS and then preserved in QIAzol lysis reagent (Qiagen, 79306) at −80°C until RNA extraction was performed using the RNeasy mini kit (Qiagen, 74106) according to the manufacturer's protocol. cDNA was synthesized using the SuperScript VILO cDNA Synthesis Kit (Invitrogen, 11754050) according to the manufacturer's recommendations. The sequences of primers used for qPCR analysis are listed in Table 1. Amplification by qPCR was performed with the QuantiTect SYBR Green PCR Kit (Qiagen, 204143) on the QuantStudio 5 (Applied Biosystems) under the cycling conditions recommended by the manufacturer.

**TABLE 1:** qPCR primer sequence.

Primer	Direction	Sequence (5′–3′)
PIK3C2A Primer Pair 1	Forward	AGGCAGCTTGAGGATTCGAC
	Reverse	GCAAGGCCTATGTGACTCCC
PIK3C2A Primer Pair 2	Forward	TCAGGCAGCTTGAGGATTCG
	Reverse	AGCAAGGCCTATGTGACTCC
β‐actin	Forward	AAGTGTGACGTGGACATCCG
	Reverse	GATGTGACAGCTCCCCACAC
HPRT	Forward	AGATGGTCAAGGTCGCAAGC
	Reverse	ATGACACAAACATGATTCAAATCCC
RPL19	Forward	CCGCTTACCTATGCCCATGT
	Reverse	AAATCGCCAATGCCAACTCC

*Note:* Primers used for qPCR analysis are listed here. β‐actin, HPRT, and RPL19 are used as internal controls for qPCR analysis.

### 2.11 Statistical Analysis

Statistical analysis was performed by using GraphPad Prism 10. One‐way ANOVA was used to compare means of different treatment groups.

## 3 Results

**FIGURE 1:**
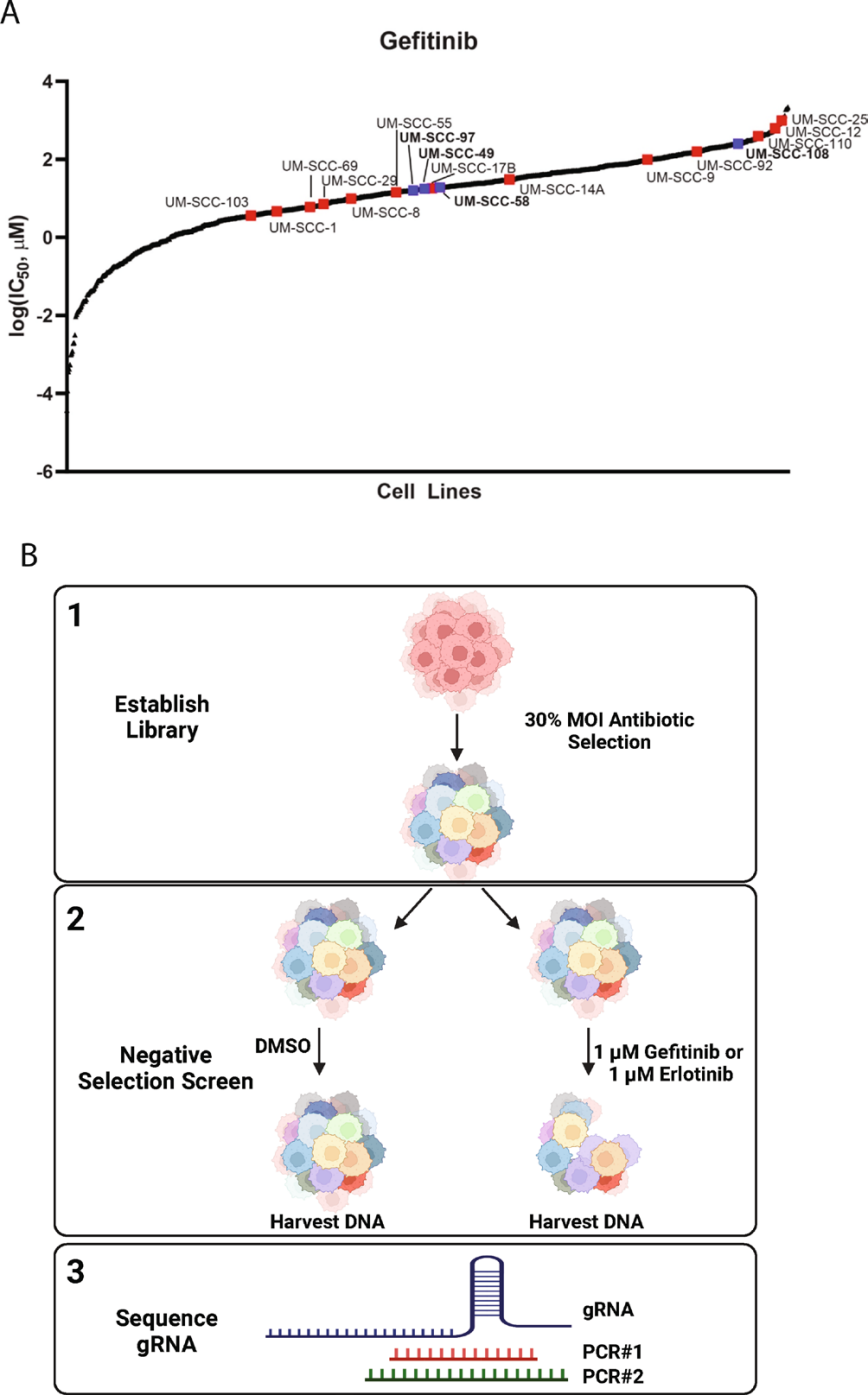
Identification of UM‐SCC cell lines that are resistant to gefitinib for use in CRISPR screening. (A) Response of different cell lines to EGFR inhibitor gefitinib. Y‐axis shows the log10 value of IC50 (μM). Black dots are the cell lines downloaded from The Genomics of Drug Sensitivity in Cancer Project. Red dots are UM‐SCC cell lines used for the resazurin viability assay. Blue dots are cell lines used for CRISPR screen. UM‐SCC cell lines' IC50 were determined from the mean ± standard deviation (SD) of at least quadruplicate measurements for each treatment and cell line. (B) Schematics of negative‐selection CRISPR screening workflow. *Source:* This figure was created with BioRender.com. [Color figure can be viewed at wileyonlinelibrary.com]

To identify mediators of resistance to EGFR‐targeted therapy in HNSCC, we performed genome‐ and kinome‐wide CRISPR/Cas9 screens in HNSCC cell lines. We first performed resazurin cell viability assays to select HNSCC cell lines resistant to the EGFR inhibitor gefitinib. Based on their gefitinib IC50 values, we chose four patient‐derived HNSCC cell lines UM‐SCC‐49, UM‐SCC‐58, UM‐SCC‐97, and UM‐SCC‐108 for subsequent CRISPR knockout screen (Figure 1A). Among these four gefitinib‐resistant cell lines, UM‐SCC‐108 exhibits the highest resistance, whereas the other three are expected to respond to gefitinib. Figure 1B shows the schematic of the negative‐selection CRISPR screen to identify genetic knockouts that confer sensitivity to EGFR inhibitor treatment. We transduced UM‐SCC‐49, UM‐SCC‐58, and UM‐SCC‐108 with established GeCKO libraries, followed by puromycin selection, and then challenged the transduced cells with either DMSO, 1 μM gefitinib, or 1 μM erlotinib. After drug treatment, we isolated genomic DNA from the surviving cells and quantified the gRNAs in each treatment group by next‐generation sequencing. We maintained a broad library diversity coverage across conditions, except for the UM‐SCC‐49 GeCKO v2A library treated with erlotinib (Figure S1A). We first tried to identify significantly depleted genes common to all three cell lines after EGFR inhibitor treatments. To our surprise, no genes were found to overlap across the three cell lines for both gefitinib and erlotinib treatments (Figure S1B). Similarly, there was minimal overlap when analyzing gefitinib‐only or erlotinib‐only results between all three cell lines (Figure S1C,D).

Since we identified few genes in common from all GeCKO screens, we then used the Human Kinase CRISPR Knockout Library as a parallel approach to provide additional statistical support to prioritize candidates. Although the Human Kinase CRISPR Knockout Library has fewer gene targets for knockout than the GeCKO libraries, it provides a greater number of gRNAs per gene to increase the statistical power of the analysis (Table 2). UM‐SCC‐49, UM‐SCC‐97, and UM‐SCC‐108 were transduced with this kinase library and were treated with either DMSO, 1 μM gefitinib, or 1 μM erlotinib after pool expansion. UM‐SCC‐58 was excluded from this screen and further validation due to its challenging growth properties. Sequencing analysis of the surviving cell population following drug treatments showed the library diversity content was above 80% coverage for the majority of the conditions (Figure S2A).

**TABLE 2:** CRISPR library statistics.

	GeCKO v1	GeCKO v2 (A or B)	Kinase
Gene targets	18 080	19 050	684
miRNA targets	0	1864	0
gRNA/gene	~3–4	3	~9
Cells/treatment	30 million	30 million	3 million

*Note:* This table depicts the number of targets, gRNAs, and cells plated per treatment to maintain library coverage for each of the three CRISPR libraries used.

**FIGURE 2:**
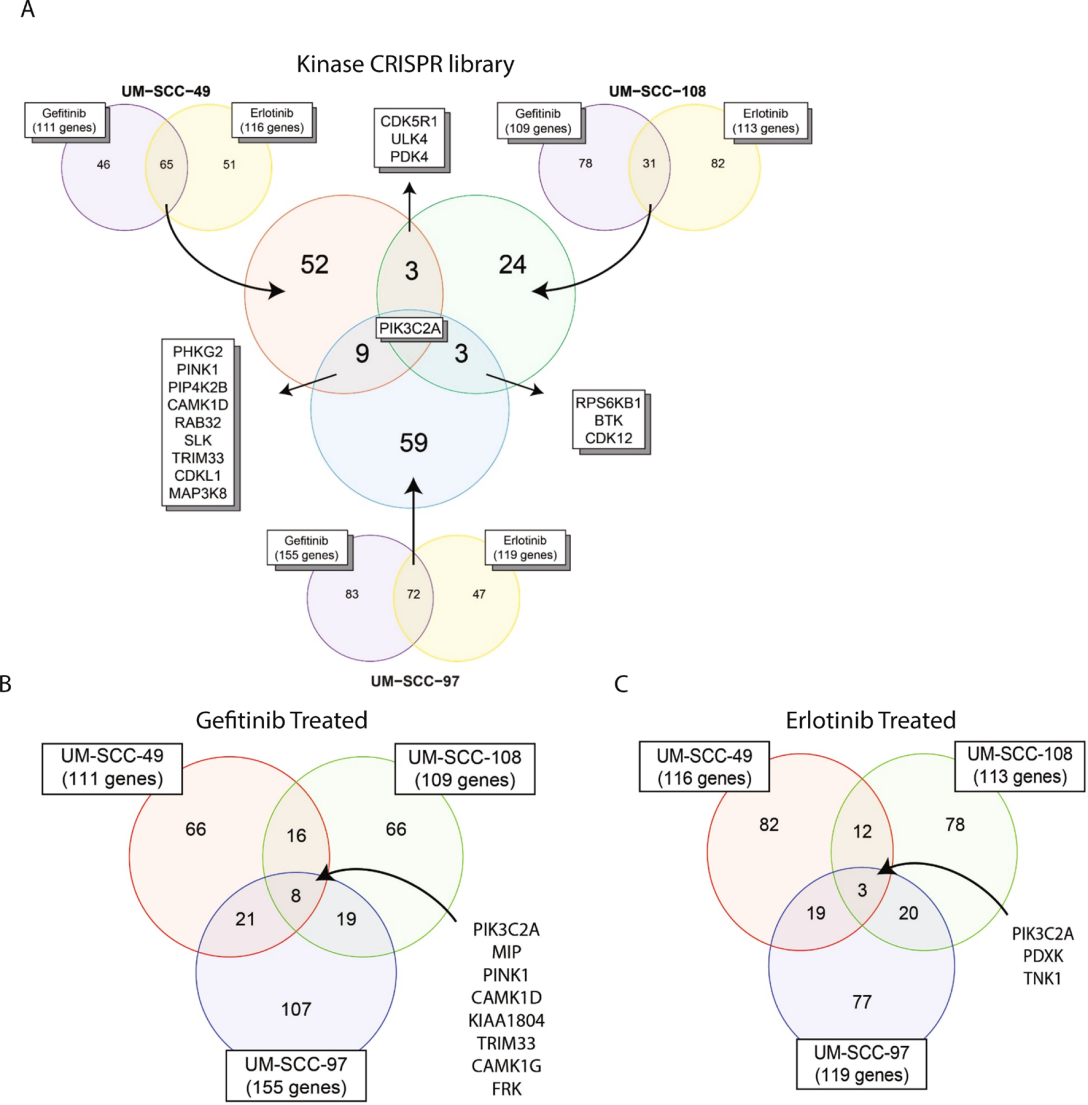
Kinase library CRISPR screen identified *PIK3C2A* as an overlapped gene that was significantly depleted across different cell lines. Venn diagram of the significantly depleted genes (*p* value ≤ 0.05) between cell lines for gefitinib and erlotinib (A), gefitinib alone (B), or erlotinib alone (C) for each Kinase library screen as indicated. [Color figure can be viewed at wileyonlinelibrary.com]

We then used the MAGeCK algorithm to identify significant gRNA and gene depletion events in the gefitinib or erlotinib treated cells compared to their respective controls. For UM‐SCC‐49, 111 genes were significantly depleted in the gefitinib treatment and 116 genes in the erlotinib treatment (*p* value ≤ 0.05), with 65 genes in common between both treatments. UM‐SCC‐108 had 109 significant genes in the gefitinib treatment and 113 genes in the erlotinib treatment (*p* value ≤ 0.05), with 31 genes overlapping. UM‐SCC‐97 had 155 significant genes for gefitinib and 119 genes for erlotinib (*p* value ≤ 0.05), with 72 genes in common. There were nine genes in common between UM‐SCC‐49 and UM‐SCC‐97 (*PHKG2, PINK1, PIP4K2B, CAMK1D, RAB32, SLK, TRIM33, CDKL1, MAP3K8*), three genes in common between UM‐SCC‐49 and UM‐SCC‐108 (*CDK5R1, ULK4, PDK4*), and three genes in common between UM‐SCC‐108 and UM‐SCC‐97 (*RPS6KB1, BTK, CDK12*). Six candidate genes (*PIK3C2A*, *CDK12*, *PINK1*, *PDXK*, *MARVELD3*, *FGFR3*) with significance from both GeCKO and Kinase library screens are listed in Table 3. We also noted eight genes overlapping between gefitinib treatment across all three cell lines (*PIK3C2A, MIP, PINK1, CAMK1D, KIAA1804, TRIM33, CAMK1G, FRK*), and three for erlotinib treatment (*PIK3C2A, PDXK, TNK1*) (Figure 2B,C). *PIK3C2A* was the only significantly depleted gene for both gefitinib and erlotinib treatments across all three lines (Figure 2A). Given the increase in overlap between EGFR inhibitors and between models with the Kinase library compared to the GeCKO, these results were felt to more accurately represent possible mechanisms of EGFR inhibitor resistance.

**TABLE 3:** Significance of six nominated genes across all library screens. [Color table can be viewed at wileyonlinelibrary.com]

	Kinase libraries						GeCKO libraries					
	UM‐SCC‐49		UM‐SCC‐108		UM‐SCC‐97		UM‐SCC‐49		UM‐SCC‐108		UM‐SCC‐58	
	Gefitinib	Erlotinib	Gefitinib	Erlotinib	Gefitinib	Erlotinib	Gefitinib	Erlotinib	Gefitinib	Erlotinib	Gefitinib	Erlotinib
PIK3C2A	0.0005	0.0019	0.0006	0.0324	0.0282	0.0244	0.5458	0.9953	0.3398	0.1086	0.6822	0.0914
CDK12	0.0565	0.1755	0.0361	0.0026	0.0340	0.0301	0.0767	—	0.7889	0.3910	0.2189	0.1416
PINK1	0.0102	0.0215	0.0355	0.3252	0.0298	0.0014	0.6011	0.0353	0.1162	0.6672	0.8318	0.0458
PDXK	0.1184	0.0223	0.0767	0.0006	0.0063	0.0405	0.9624	—	0.0719	0.0131	0.1262	0.4262
MARVELD3	0.0008	0.0028	0.1082	0.0068	0.1848	0.4968	0.2190	0.7358	0.9380	0.9406	0.2563	0.4117
FGFR3	0.0110	0.0150	0.1758	0.2505	0.8514	0.9566	0.8124	0.4790	0.0789	0.1088	0.0691	0.3059

*Note:* Table of *p* values from MAGeCK output by gene for both Kinase and GeCKO library screens. *p* values that are ≤ 0.05 are colored green.

**FIGURE 3:**
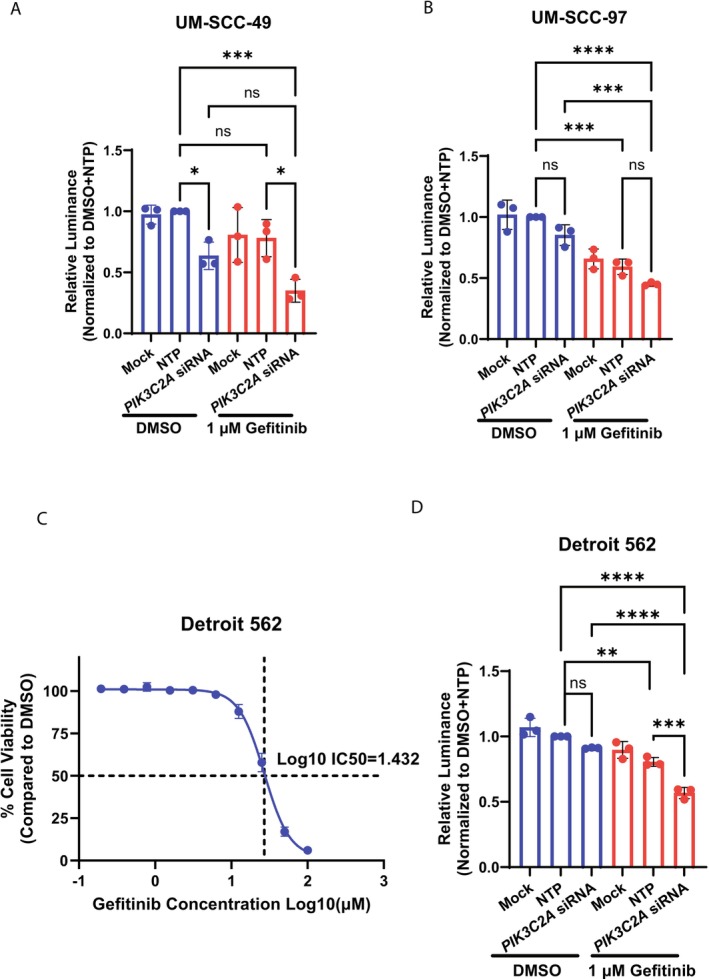
CellTiter‐Glo viability assay confirmed that *PIK3C2A* knockdown in combination with gefitinib shows better growth inhibition in HNSCC cell lines. UM‐SCC‐49 (A), UM‐SCC‐97 (B), and Detroit 562 (D) were treated with transfection reagent alone (Mock), non‐targeting control pool (NTP), *PIK3C2A* siRNA SMARTPool with either DMSO or 1 μM gefitinib. CellTiter‐Glo viability assay was applied to determine cell viability, and the fold change in viability was determined by normalizing the luminescence signal to the DMSO + NTP treatment group. Error bars are mean ± SD from three independent experiments. One‐way ANOVA was used to compare relative viability means across different treatment groups. *p < 0.05, **p < 0.01, ***p < 0.001, and ****p < 0.0001. (C) Determination of IC50 of gefitinib in Detroit 562. Detroit 562 cells were treated with increasing concentrations of gefitinib (0.2–100 μM) for 72 h. Resazurin viability assay was used to determine cell viability, and the percentage of viability was calculated by normalizing the fluorescence signal to the DMSO control group. Dose–response curve along with IC50 was calculated by using a non‐linear regression model from GraphPad Prism. Data are shown as mean ± standard SD of quadruplicate measurements from one experiment. [Color figure can be viewed at wileyonlinelibrary.com]

As *PIK3C2A* was the most recurrent candidate across the screens and may represent a potential mechanism of activation of the PI3K/mTOR signaling node, we decided to further investigate its possible role in resistance to EGFR inhibition. We hypothesized that the knockdown of *PIK3C2A* with siRNA would enhance sensitivity to the EGFR inhibitor gefitinib in resistant HNSCC cell lines. We transfected the three UM‐SCC cell lines used in the Human Kinase CRISPR Knockout Library screens with either transfection reagent alone (Mock), a NTP, or human *PIK3C2A* siRNA for 24 h. Afterward, we treated the cells with either DMSO or gefitinib for an additional 48 h. Consistent with the result of our CRISPR screen, the combination of *PIK3C2A* siRNA and gefitinib showed the lowest viability in UM‐SCC‐49 and UM‐SCC‐97. In UM‐SCC‐49, *PIK3C2A* siRNA + gefitinib significantly reduced viability compared to NTP + DMSO (*p* = 0.0005) and NTP + gefitinib (*p* = 0.0151) (Figure 3A); in UM‐SCC‐97, *PIK3C2A* siRNA + gefitinib significantly reduced viability compared to NTP + DMSO (*p* < 0.0001) and *PIK3C2A* siRNA + DMSO (*p* = 0.0003) (Figure 3B). To our surprise, the CellTiter‐Glo assay showed UM‐SCC‐108 was more sensitive to gefitinib. This is inconsistent with the resazurin assay result (Figure 1A), as gefitinib alone significantly reduced the viability (*p* < 0.0001) whereas the combination of *PIK3C2A* siRNA and gefitinib did not enhance this (Figure S3A). To extend our findings beyond the UM‐SCC cell lines and examine whether *PIK3C2A* downregulation is also effective in HNSCC models with PI3K signaling pathway alterations, we also applied the same treatment strategy to another human HNSCC cell line Detroit 562, which has a gain‐of‐function mutation (H1047R) in the *PIK3CA* gene. Detroit 562 is resistant to gefitinib and exhibits a similar gefitinib IC50 to the selected UM‐SCC cell lines (Figure 3C). In Detroit 562, the combination of *PIK3C2A* siRNA and gefitinib significantly reduced viability compared to both NTP + DMSO (*p* < 0.0001), *PIK3C2A* siRNA + DMSO (*p* < 0.0001), and NTP + gefitinib (*p* = 0.0003) (Figure 3D). Knockdown efficiency was determined by qPCR after 72 h transfection (Figure S3B). In conclusion, though the degree of benefit from a single treatment timepoint greatly varies among different cell lines, reducing *PIK3C2A* mRNA levels sensitized HNSCC cell lines to EGFR inhibition with gefitinib.

To better understand how the *PIK3C2A* mRNA downregulation sensitizes HNSCC cells to gefitinib, we examined cell cycle progression and cell apoptosis. We chose Detroit 562 for this mechanism determination as it showed the greatest sensitivity to the combination of *PIK3C2A* siRNA and gefitinib. Cell cycle flow cytometry analysis showed that gefitinib treatment reduced cell population at S‐phase, though the combination of *PIK3C2A* siRNA and gefitinib did not further reduce S‐phase population. This change is similar to a previous study from Shintani et al. that showed gefitinib reduces S‐phase population in oral squamous cell carcinoma cell lines [30]. However, our data show no significant alteration for G0/G1‐phase distribution across the different treatment groups (Figure S3C). Analysis of apoptosis by flow cytometry showed the combination of gefitinib with *PIK3C2A* siRNA had the highest apoptotic cell population relative to other control groups, though this increase was not statistically significant (Figure S3D).

## 4 Discussion

Although the overexpression of EGFR and its ligands is prevalent in HNSCC patients and correlates with tumorigenesis, EGFR inhibitors that are either FDA‐approved or under investigation showed limited efficacy as monotherapies [8, 9, 14, 16]. Given the heterogeneous genetic landscapes and the complex crosstalk among different signaling pathways, the mechanism of EGFR inhibitor resistance in HNSCC is not well understood to date. Previous studies have shown that the PI3K and EGFR signaling contribute to the inhibition resistance of each other in HNSCC [31, 32, 33]. Our team's previous work also demonstrated that the combination of the gefitinib and the PI3K inhibitor HS‐173 showed potential synergistic growth inhibition in some HNSCC models [23]. Boehm et al. [34] demonstrated that the triple inhibition of EGFR, signal transducer and activator of transcription‐3 (STAT3), and Bcl‐X_L_ had better antitumor effects in HNSCC cell lines. Additionally, they also showed that the dual inhibition of EGFR and STAT3 had enhanced antitumor effects in vivo. These studies detail the advantages of combination strategies, as well as the importance of identifying key mediators of EGFR inhibitor resistance in HNSCC.

In this paper, we introduced a genome‐wide, unbiased approach to identify mediators of EGFR inhibitor resistance in HNSCC. We used two CRISPR screening libraries to identify *PIK3C2A* as a novel key mediator of resistance to the EGFR inhibitor gefitinib in HNSCC cell lines. We further validated this finding by showing that *PIK3C2A* knockdown increased the gefitinib sensitivity in gefitinib‐resistant HNSCC cells. Interestingly, the nature of our screening strategy, which tested a therapeutic multiple times, appears to have identified hits with modest effect sizes in single time‐point experiments that may multiply over time and eventually cause a larger effect.

Human PI3K‐C2α, encoded by the *PIK3C2A* gene, belongs to class II PI3Ks. Domin et al. [35] firstly colonized and characterized PI3K‐C2α, showing this catalytic subunit is ubiquitously expressed across most human tissues while having lower sensitivity to the PI3K inhibitors wortmannin and LY294002 compared to class I PI3K. This result indicates PI3K‐C2α may act on other signaling pathways instead of the canonical PI3K/AKT pathway to drive EGFR inhibitor resistance in HNSCC. Indeed, unlike the characterized oncoprotein class I PI3Ks, the role of PI3K‐C2α in cancer remains controversial and could be tissue‐ and tumor‐specific. Elis et al. [36] reported a 75% downregulation of PI3K‐C2α was sufficient to induce cell death by apoptosis in HeLa cells, and the viability of other carcinoma cell lines from various organs also decreases with PI3K‐C2α inhibition. Ng et al. [37] conducted a similar study in hepatocellular carcinoma (HCC), showing that *PIK3C2A* siRNA reduced HCC cell proliferation through caspase‐3‐mediated apoptosis. In contrast, Dai et al. [38] showed that lower *PIK3C2A* expression was associated with higher risk, poorer prognosis, and shorter overall survival in patients with clear‐cell renal‐cell carcinoma. A more recent study from Gulluni et al. [39] reported a more detailed function of PI3K‐C2α in breast cancer: PI3K‐C2α protein acts as a scaffold protein to maintain spindle stability, while its downregulation inhibited breast tumor proliferation and onset during early stages but promoted tumor growth at later stages due to mitotic checkpoint defects. Additionally, the compromised spindle stability resulting from PI3K‐C2α loss increased sensitivity to taxane‐based therapy in breast cancer models. Our study is the first to demonstrate PI3K‐C2α acts as a driver of EGFR inhibitor resistance in HNSCC, as reducing *PIK3C2A* transcript levels led to growth inhibition and increased sensitivity to the EGFR inhibitor gefitinib, especially in long‐term culture models. The similarities and the differences of PI3K‐C2α functions observed between our study and others suggest further studies are warranted to explore the possible tumor‐specific role of PI3K‐C2α.

We also investigated the potential mechanisms underlying the enhanced growth inhibition from combined EGFR and PI3K‐C2α inhibition. While a significant increase in apoptotic cells was not observed overall, Detroit 562 showed a trend toward increased apoptosis in the combination treatment group. Cell cycle flow cytometry analysis showed EGFR inhibition alone was sufficient to reduce the cell population going through S‐phase, while adding PI3K‐C2α inhibition did not further enhance this cell cycle dysregulation. Given that our CRISPR screens achieved the *PIK3C2A* knockout and the treatment was a multi‐dose EGFR inhibitor over the longer term, our siRNA knockdown followed short‐term EGFR inhibition may not be sufficient to reveal a clear mechanism. Additionally, apoptosis alone may be insufficient, and exploring other cell death mechanisms such as DNA damage, necrosis, and ferroptosis could provide a better understanding as to how this combination overcomes EGFR inhibitor resistance in HNSCC. Moreover, animal experiments, either by implanting *PIK3C2A* downregulated tumors or by using PI3K‐C2α specific inhibitors, are warranted to determine whether PI3K‐C2α inhibition can sensitize resistant HNSCC tumors to EGFR inhibition.

In summary, we performed negative‐selection CRISPR screens followed by RNA interference to identify and validate *PIK3C2A* downregulation driving sensitivity to EGFR inhibition in HNSCC. Our work proved this class II PI3K member, PI3K‐C2α, functions as an EGFR inhibitor resistance mediator in HNSCC. This study can provide insights for future studies to better understand EGFR inhibitor resistance in HNSCC. Our work may also inform the development of specific PI3K‐C2α inhibitors as well as novel combination strategies for HNSCC patients.

## Disclosure

The authors have nothing to report.

## Conflicts of Interest

The authors declare no conflicts of interest.

## Supporting information


**Data S1:** hed70048‐sup‐0001‐Supinfo1.docx.

**Figure S1:** hed70048‐sup‐0002‐FigureS1.pdf.

**Figure S2:** hed70048‐sup‐0004‐FigureS2.pdf.

**Figure S3:** hed70048‐sup‐0005‐FigureS3.pdf.

## Data Availability

The data that support the findings of this study are available from the corresponding author upon reasonable request.
