# Supplementary material for: CRISPR Screens Identify PIK3C2A as a Novel Mediator of EGFR Inhibitor Resistance in Head and Neck Squamous Cell Carcinoma
Source: Head Neck. 2025 Sep 23;48(2):486–95. doi: 10.1002/hed.70048 (PMC12797014; doi:10.1002/hed.70048)
Supplement: Supplementary file 1 — Data S1: hed70048‐sup‐0001‐Supinfo1.docx. [file HED-48-486-s003.docx]

**Supplemental Methods**

**GeCKO Library Preparation.** To maintain the coverage of the GeCKO library, 130 µg of genomic DNA was used to PCR amplification of the gRNA sequence using the Herculase ii Fusion DNA Polymerase (Agilent # 600675). The following primers were used with 10 µg input DNA for 13 reactions for amplification:

PCR #1 Fwd: AATGGACTATCATATGCTTACCGTAACTTGAAAGTATTTCG

PCR #1 Rev: GGTCTTGAAAGGAGTGGGAATTGGCTCCGGTGCCCGTCAG

The second round PCR reactions were set up with 5 µL of combined 13 reactions along with the following primers:

PCR #2 Fwd: AATGATACGGCGACCACCGAGATCTACACTCTTTCCCTACACGACGCTCTTCCGATCT(1-9bp stagger)AAGTAGAGtcttgtggaaaggacgaaacaccg

PCR #2 Rev: CAAGCAGAAGACGGCATACGAGATTCGCCTTAGTGACTGGAGTTCAGACGTGTGCTCTTCCGATCTataacggactagccttattttaac

Illumina adapters are in uppercase. The forward primer contains the TruSeq Universal Adapter. The reverse primer includes Illumina P7, 8bp index and multiplexing PCR primer 2.0. The sequence of an 8bp barcode is underlined. The priming sites of lentiviral construct are in lower case.

Gel Extraction PCR Purification Kit (Qiagen) was used to extract and purify the PCR prodcut before submission to the University of Michigan DNA Sequencing Core for sequencing with Illumina HiSeq 2500 High-Output with V4 Kit.

**Kinase Library Preparation.** To preserve coverage of the Kinase Library, 12 µg of DNA was used to PCR amplify the gRNA sequence using the Herculase ii Fusion DNA Polymerase (Agilent # 600675). 2 reactions with 6 µg input DNA were amplified with the following primers:

PCR #1 Forward : AATGGACTATCATATGCTTACCGTAACTTGAAAGTATTTCG

PCR #1 Reverse: CTCGATTAATTAAGGTTGCTCACTTGTCGACTAATGC

The two reactions were then combined, and 5 µL were used to set up the second round of PCR reactions. PCR #2 primers are same as listed above in the GeCKO Library Preparation section, as this adds on Illumina adaptor sequences and barcodes.

The PCR products were gel extracted and purified using Gel Extraction PCR Purification Kit (Qiagen). Samples were then submitted to the University of Michigan DNA Sequencing Core for sequencing with Illumina MiSeq V3 Kit.

**CRISPR libraries transduction.** UM-SCC lines were transduced with the Human GeCKO CRISPR knockout pooled library, either version 1 (Addgene plasmid #49535) or version 2 (Addgene plasmid #52961) which were gifts from either Feng Zhang, or the Human Kinase Lentiviral CRISPR Pool (Sigma Aldrich HKCRISPR). Cells were transduced at a multiplicity of infection of 0.3 to prevent more than one gRNA integrating into a cell. After 7 days of puromycin selection, the cells were expanded and seeded to preserve 300x coverage of gRNA libraries for each treatment (**Table 1**). At the end of treatment, genomic DNA was isolated from the surviving cells by using Gentra Puregene Cell Kit (Qiagen).

**Analysis of CRISPR libraries.** Reads were demultiplexed based on barcode and mapped to the corresponding reference library by using an in-house python script. gRNA counts were entered into Model-based Analysis of Genome-wide CRISPR/Cas9 knockouts (MAGeCK, v0.5.2) to determine significant gRNAs and genes [1]. Genes with an α-RRA score of ≤0.05 were advanced to GSEA analysis. GSEA was then performed using the Molecular Signatures Database (v5.1) with the GSEA3.0.jar module to identify overlap with “Hallmark”, “C3_motif”, “Go-BP” and “Oncogene” gene set databases. Analysis was performed with 1000 permutations and gene sets with false discovery rate of less than 0.05 were considered significant (max of 20 gene sets per reference database) as described [2,3] and advanced for network analysis of representative and recurrent gene sets using the Cytoscape_v3.7.1 desktop module.

**Venn diagrams**. Venn diagrams were modified from the output of Galaxy’s Venn Diagram program [4].

**References**

1. Li W, Xu H, Xiao T, et al. MAGeCK enables robust identification of essential genes from genome-scale CRISPR/Cas9 knockout screens. *Genome biology.* 2014;15(12):554.

2. Subramanian A, Tamayo P, Mootha VK, et al. Gene set enrichment analysis: a knowledge-based approach for interpreting genome-wide expression profiles. *Proceedings of the National Academy of Sciences.* 2005;102(43):15545-15550.

3. Mootha VK, Lindgren CM, Eriksson K-F, et al. PGC-1α-responsive genes involved in oxidative phosphorylation are coordinately downregulated in human diabetes. *Nature genetics.* 2003;34(3):267-273.

4. Cock PJA, Grüning BA, Paszkiewicz K, Pritchard L. Galaxy tools and workflows for sequence analysis with applications in molecular plant pathology. *PeerJ.* 2013;1:e167-e167.
