# Supplementary figures and images for: CRISPR Screens Identify PIK3C2A as a Novel Mediator of EGFR Inhibitor Resistance in Head and Neck Squamous Cell Carcinoma
Source: Head Neck. 2025 Sep 23;48(2):486–95. doi: 10.1002/hed.70048 (PMC12797014; doi:10.1002/hed.70048)

Figure 1 Sup

A

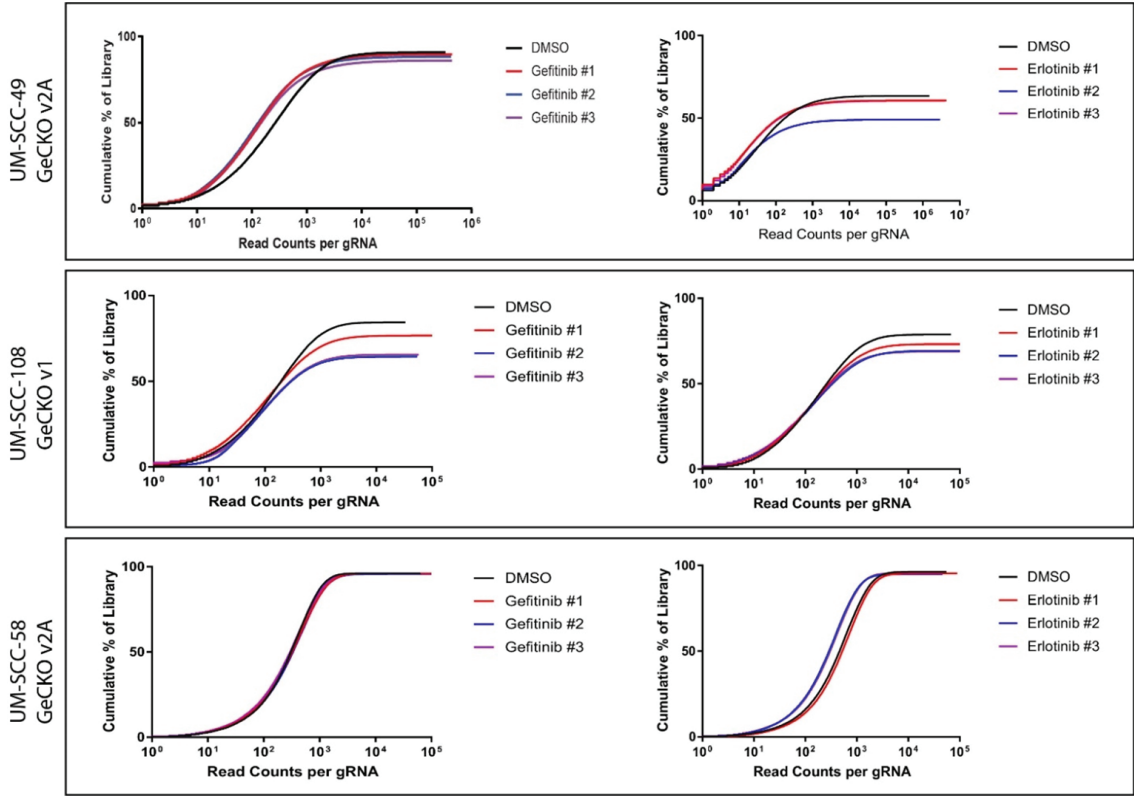

B

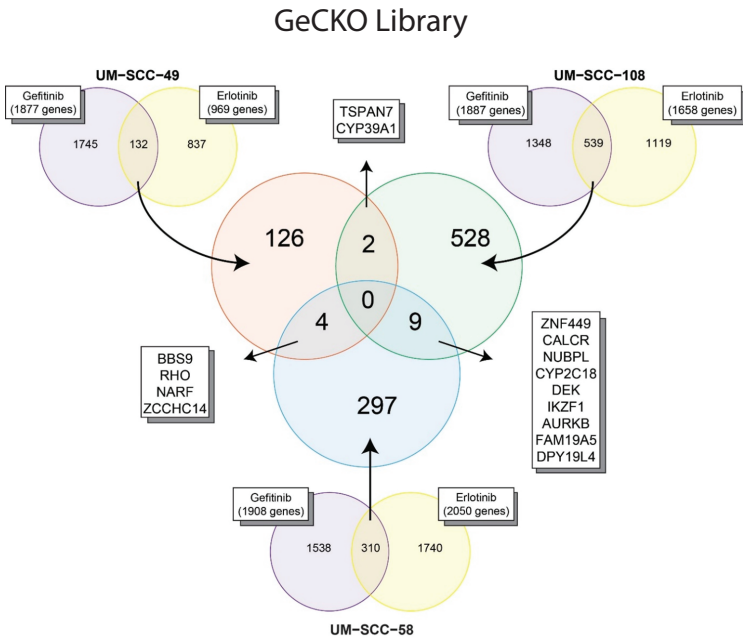

C

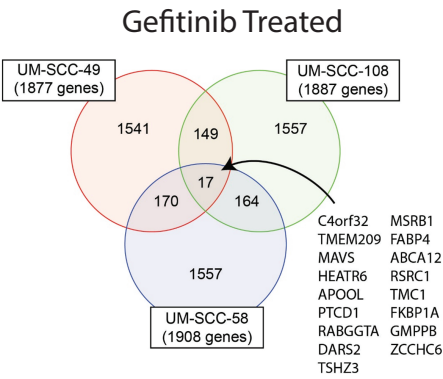

D

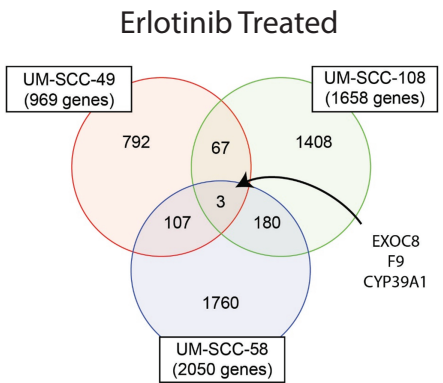

Supplement: Supplementary file 2 — Figure S1: hed70048‐sup‐0002‐FigureS1.pdf. [file HED-48-486-s004.pdf]

Figure 2 Sup

A

Kinase CRISPR library

UM-SCC-49

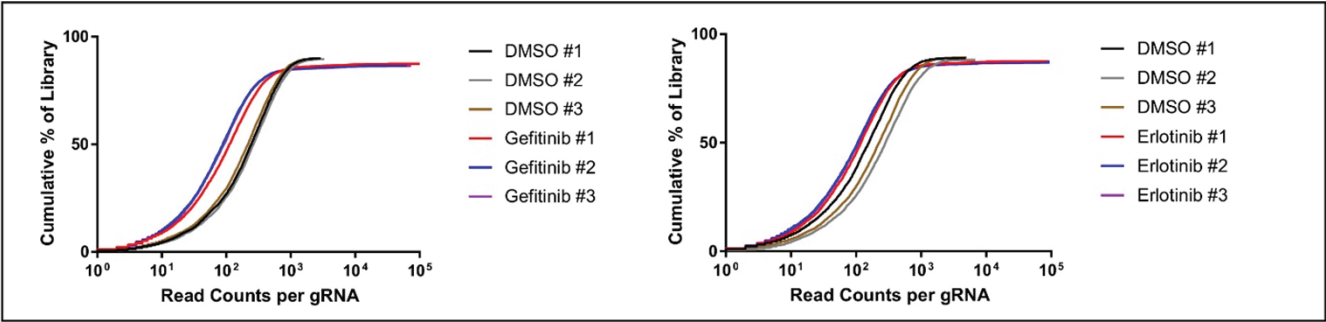

UM-SCC-108

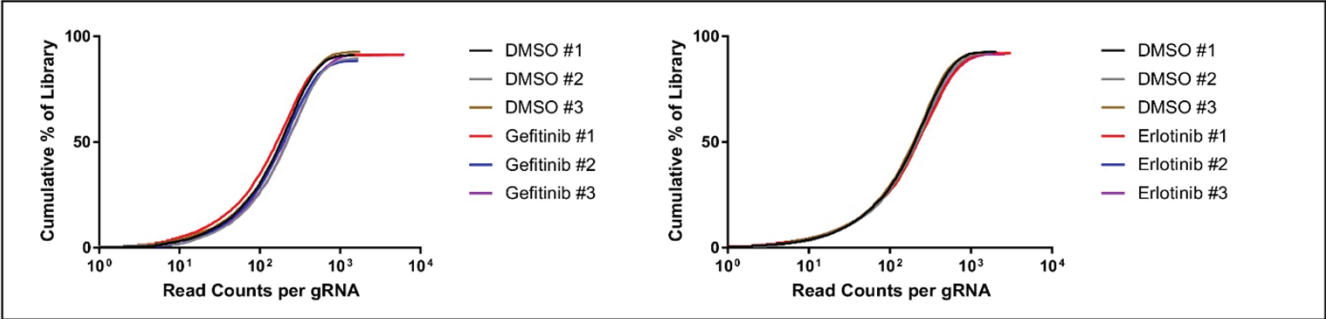

UM-SCC-97

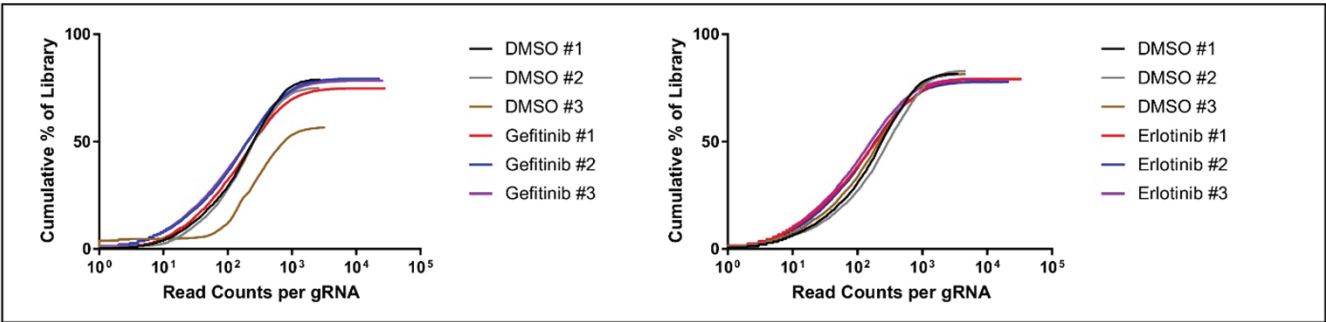

Supplement: Supplementary file 3 — Figure S2: hed70048‐sup‐0004‐FigureS2.pdf. [file HED-48-486-s002.pdf]

A

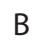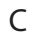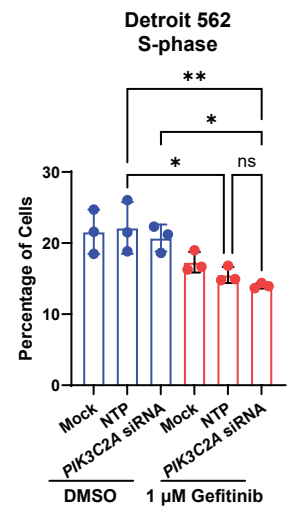

D

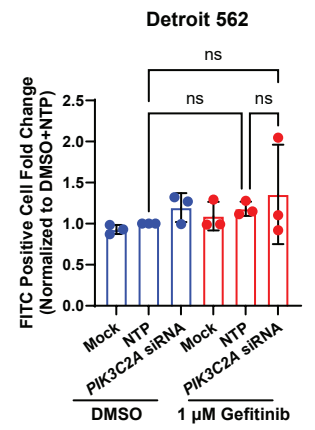

Supplement: Supplementary file 4 — Figure S3: hed70048‐sup‐0005‐FigureS3.pdf. [file HED-48-486-s001.pdf]
